# An in vitro study on the bond strength between composite resin and porcelain using different surface treatment and bonding methods

**DOI:** 10.1111/eos.70014

**Published:** 2025-05-05

**Authors:** Mina Aker Sagen, Bjørk Eriksen, Kaja Moland, Bjørn Einar Dahl

**Affiliations:** ^1^ Nordic Institute of Dental Materials Oslo Norway; ^2^ Institute for Clinical Dentistry, Faculty of Odontology University of Oslo Oslo Norway

**Keywords:** ceramic, chipping, fixed prosthodontics, repair, shear forces

## Abstract

The study aimed to determine the effect of different surface treatments and bonding methods on bond strength between composite resin and feldspar ceramic, focusing on the interplay between these variables for enhanced clinical outcomes of chipping repair. Two surface treatments (hydrofluoric acid [HF] etching and surface roughening using a diamond disc) were investigated alongside two bonding methods utilizing either a universal adhesive (Scotchbond Universal, 3M) or a separate silane primer and adhesive (Intraoral Repair Kit, BISCO). Bond strength was measured using shear forces, and light microscopy was used to analyse the fracture mode. The findings indicate that the bonding method plays a more significant role than surface treatment for increasing bond strength since bond strength was similar following either roughening or HF acid etching. The separate silane primer and adhesive demonstrated superior bond strength over the universal adhesive, despite both containing silane, and this was attributed to differences in silane monomer stability. Selecting an appropriate bonding method, specifically using separate silane primer, enhances bond strength in composite repairs. This approach may mitigate the known risks associated with HF and addresses clinical challenges by optimizing the bond stability between feldspathic porcelain and composite resin.

## INTRODUCTION

The combination of metal and feldspar porcelain has been utilized for indirect restorations since the 1950s', with both single and multi‐unit fixed dental prostheses (FDPs) still being produced using this combination of materials [1]. In the 1990s, zirconia emerged as an alternative core material, replacing metal in many cases [2]. Despite the well‐documented and widespread application of FDPs as indirect restorations, this treatment is not devoid of complications [3, 4]. Chipping of feldspar porcelain remains one of the challenges associated with bilayered restorations, and occurs more frequently when zirconia, rather than metal, is used as a core material [2, 4, 5]. Factors contributing to this phenomenon include trauma, improper design, material defects, and occlusal interferences [6].

Chipping typically manifests as either a cohesive fracture confined to the veneering feldspar porcelain or an adhesive fracture between the feldspar porcelain and the core material [2]. It is essential to address chipping, as rough fracture surfaces can heighten the likelihood of subsequent fractures and contribute to increased wear on opposing teeth. [3]. Also, chipping of restorations in the aesthetic zone typically requires intervention. Treatment options include intraoral repair or complete restoration revision, depending on the severity of the chipping. Generally, minor chippings can be managed by intraoral repair, whereas extensive fractures necessitate restoration revision. Intraoral repair with composite resin offers a time‐efficient, minimally invasive, and cost‐effective alternative if the prognosis is favourable [7, 8]. Successful repair relies on micromechanical roughness and chemical bonding between composite resin and the ceramic fracture surface [3, 8].

The most common method for repairing feldspar porcelain involves hydrofluoric acid (HF) etching, creating micro roughness on the surface and exposing bonding sites for primer and/or adhesive [7]. Despite the effectiveness of HF, its high toxicity poses clinical challenges. Even at low concentrations, HF can damage mucous membranes, with symptoms of exposure not becoming evident until several hours later [9]. Consequently, intraoral HF use mandates rubber dam isolation and meticulous handling. Alternatives to HF are roughening of the ceramic surface using a dental diamond bur and surface abrasion using airborne particles or different types of lasers; although these methods do not have the toxicity of HF, the bond strength achieved is variable [10, 11].

Chemical bonding between composite resin and feldspar porcelain is established by application of an adhesive on the repair surface [7]. In many cases of repair, silane is applied as a separate primer prior to the adhesive. This is because silane can facilitate bonding by forming covalent bonds with inorganic silica particles in ceramics and with hydrophobic resin polymers in composites [12]. The effect of silane on increasing the bond strength between feldspar porcelain and composite resin is comprehensively documented [11].

Universal adhesives, designed to bond to various materials, including feldspar porcelain and composite resin, often contain silane in addition to other monomers. This makes them suitable for repair purposes with a less time‐consuming protocol than necessary when using separate primer and adhesive [13, 14]. However, challenges related to the instability of silane in universal adhesives because of a suboptimal pH level have been reported, which might affect its bond strength to feldspar porcelain and composite resin [15].

With different combinations of surface treatment methods and chemical bonding available for ceramic repair, the procedure step with the most significant effect on bond strength should be outlined. Therefore, the aim of this in vitro study was to explore the impact of pretreatments of the fracture surface on the bond strength between feldspar porcelain and composite resin. The null hypothesis was that there is no difference in bond strength between feldspar porcelain and composite resin when two different surface treatments and two different bonding methods are used.

## MATERIAL AND METHODS

### Specimen preparation and grouping

Forty square‐shaped (5 mm × 5 mm × 2 mm) specimens were prepared from feldspar porcelain powder and modelling liquid (Initial Zr‐FS, GC Europe N.V.). Specimens were embedded in acrylic resin with the surfaces to be bonded to composite resin exposed. The surface of all specimens were ground using a rotating P1200 diamond grinding disc for uniformity, after which they were steam‐cleaned and dried using compressed oil‐free air. Further, specimens were randomly divided into four experimental groups based on surface treatment and bonding method (Table 1). An overview of the products used, and their composition, is shown in Table 2. The grit (P220) of the rotating diamond disc used for roughening the feldspar porcelain surface was selected to correspond to a medium‐grit dental bur (blue colour code) according to ISO [16]. The ceramic surface was etched with HF for 90 s, according to the manufacturer's (BISCO) instructions for use.

**TABLE 1:** The four test groups.

Group	Method
R‐UA	Roughening (P220 rotating diamond disc) + universal adhesive
R‐SA	Roughening (P220 rotating diamond disc) + silane primer + adhesive
E‐UA	Etching (9.5% hydrofluoric acid for 90 s) + universal adhesive
E‐SA	Etching (9.5% hydrofluoric acid for 90 s) + silane primer + adhesive

**TABLE 2:** Overview of products used and their composition.

Product name and manufacturer	Product composition
Porcelain etchant, BISCO	9.5% hydrofluoric acid, water, sodium fluoride [17]
Intraoral Repair Kit, BISCO	Porcelain primer: acetone, TMSPM [18] Porcelain bonding resin: UDMA, Bis‐GMA, TEGDMA, THFMA, TMPTMA [19]
Scotchbond Universal Adhesive, 3M	BADGE‐DMA, HEMA, DDM, PAMA, DMAEMA, MEK, silane‐treated silica, ethanol, water, camphorquinone, acrylic acid [20]
Filtek Supreme XTE Universal Restorative A3, 3M	Bis‐GMA, UDMA, PEGDMA, ceramic fillers, camphorquinone, pigments [21]

Abbreviations: BADGE‐DMA, bisphenol A diglycidyl ether dimethacrylate; Bis‐GMA, bisphenol A‐glycidyl methacrylate; DDM, n‐dodecyl‐β‐D‐maltoside; DMAEMA, 2‐(dimethylamino)ethyl methacrylate; HEMA, 2‐hydroxyethyl methacrylate; MEK, methyl ethyl ketone; PAMA, poly alkyl methacrylate; PEGDMA, poly(ethylene glycol) dimethacrylate; TEGDMA, triethylene glycol dimethacrylate; THFMA, tetrahydrofurfuryl methacrylate; TMPTMA, trimethylolpropane trimethacrylate; TMSPM, 3‐(trimethoxysilyl)propyl methacrylate; UDMA,  urethane dimethacrylate.

After surface treatment and application of adhesive, composite resin rods with a diameter of 2.38 mm were built up using a bonding mold clamp and a bonding mold insert (Ultradent), conforming to ISO standard 29,022:2013 [22]. Following buildup, the specimens were stored in distilled water at 37°C for 24 h, followed by thermal cycling (5000 cycles at 5°C and 55°C) and subsequent storage in distilled water at 37°C until testing.

### Bond‐strength testing

Bond strength was tested using a universal mechanical testing machine (zwickiLine Z5.0, Zwick/Roell), applying shear force (1 mm/min) until fracture. The applied force at break (N) was recorded, and bond strength (MPa) was calculated.

### Fracture mode

Fracture surfaces were examined under a light microscope, and fractures were characterized as adhesive (between feldspar porcelain and composite resin), cohesive in feldspar porcelain, or cohesive in composite resin.

### Statistical analysis

The number of specimens in each group (*n* = 10) was selected based on a comparable study regarding material and method [23]. Data were analysed in STATA/SE version 18 (StataCorp) for estimation of mean, median, and standard deviation values. The Shapiro‐Wilks test was used to evaluate if the bond‐strength data followed a normal distribution. As the data were found to follow a non‐normal distribution, the Wilcoxon rank‐sum test was used to compare bond‐strength values across the groups.

## RESULTS

### Shear bond strength

**FIGURE 1:**
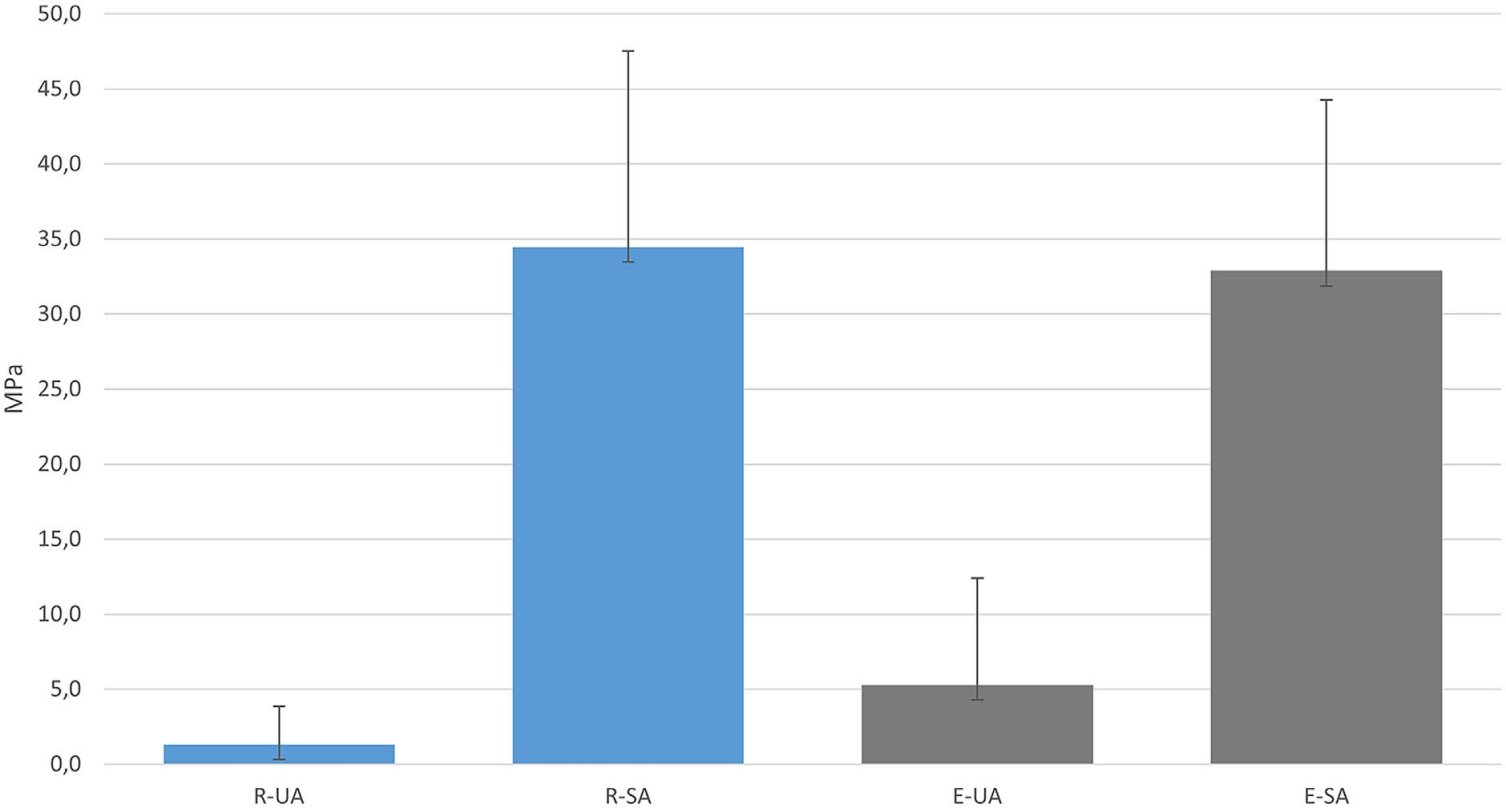
Shear bond strength analyses of the four test groups. The groups in which silane primer and adhesive (SA) were used had significantly higher bond strength than the groups in which universal adhesive (UA) was used. Results are given as mean and SD, in MPa. E‐SA, etching (9.5% hydrofluoric acid for 90 s) + silane primer + adhesive; E‐UA, etching (9.5% hydrofluoric acid for 90 s) + universal adhesive; R‐SA, surface roughening (P220 rotating diamond disc) + silane primer + adhesive; R‐UA, surface roughening (P220 rotating diamond disc) + universal adhesive.

Seven (of 10) specimens in the group treated with surface roughening and a universal adhesive (R‐UA) and five (of 10) specimens in the group treated with HF etching and universal adhesive (E‐UA) debonded during thermal cycling and therefore received a bond‐strength value of 0 MPa. The statistical analyses showed no significant difference (*p* > 0.05) in bond strength between groups treated with roughening or with etching with HF, provided that the same adhesive protocol was followed (i.e., group R‐UA vs. group E‐UA or surface roughening plus silane primer and adhesive [R‐SA] vs. HF etching plus silane primer and adhesive [E‐SA]). However, a significantly higher bond strength was found when using a separate silane primer and adhesive than when using a universal adhesive (i.e., group R‐SA vs. group R‐UA and group E‐SA vs. group E‐UA) (Figure 1).

### Fracture mode

**FIGURE 2:**
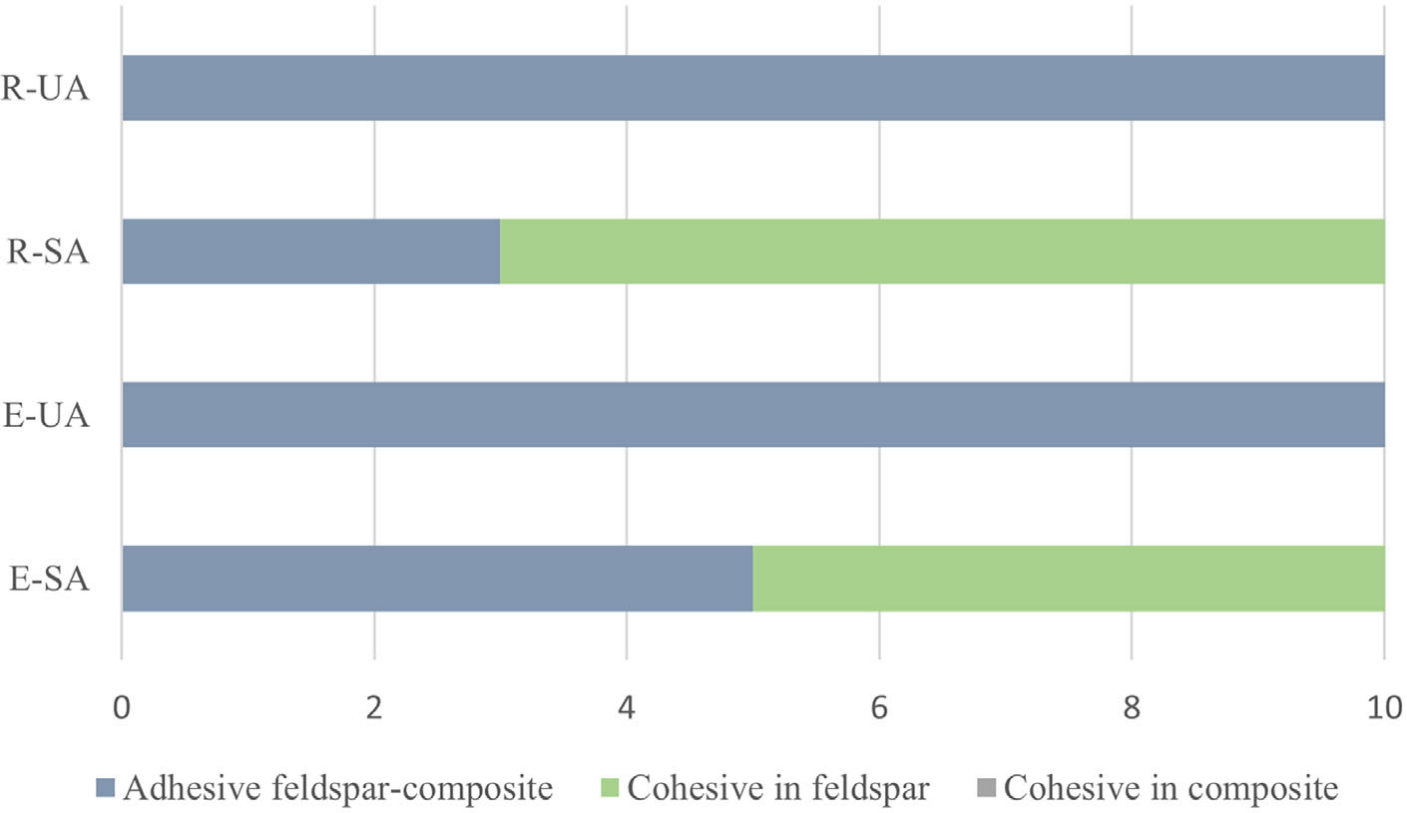
Distribution of fracture mode in the four test groups. Cohesive fracture in composite resin was not observed. E‐SA, etching (9.5% hydrofluoric acid for 90 s) + silane primer + adhesive; E‐UA, etching (9.5% hydrofluoric acid for 90 s) + universal adhesive; R‐SA, surface roughening (P220 rotating diamond disc) + silane primer + adhesive; R‐UA, surface roughening (P220 rotating diamond disc) + universal adhesive.

**FIGURE 3:**
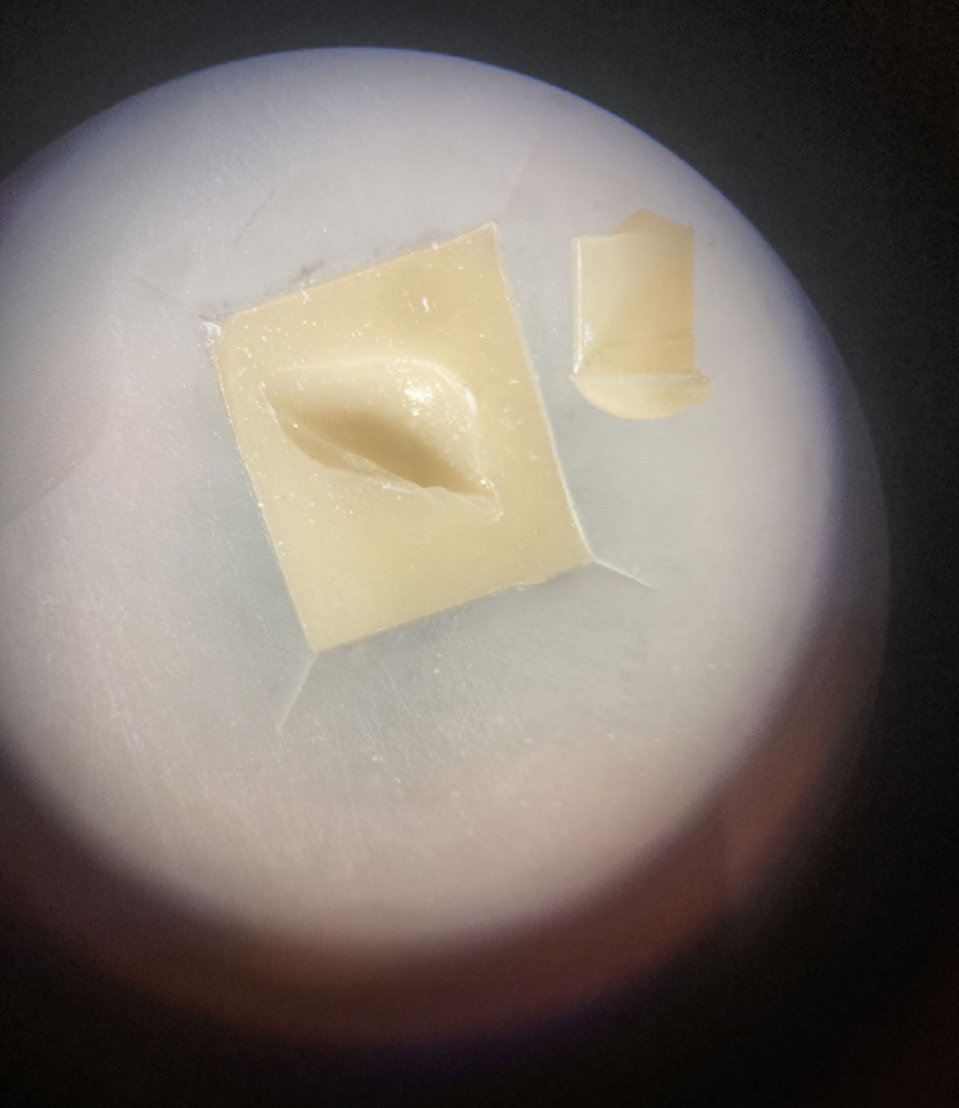
Image showing a cohesive fracture in feldspar porcelain, which was only observed for test groups in which separate silane primer and adhesive (R‐SA and E‐SA) were applied. E‐SA, etching (9.5% hydrofluoric acid for 90 s) + silane primer + adhesive; R‐SA, bur roughening (P220 rotating diamond disc) + silane primer + adhesive.

Inspection of the fracture surfaces using light microscopy revealed that specimens which debonded during thermal cycling had adhesive fractures between composite resin and feldspar porcelain This was also the dominating fracture mode for groups R‐UA and E‐UA (i.e., when a universal adhesive was applied). For the two groups in which a separate silane primer and adhesive were applied, a high frequency of cohesive fractures in porcelain occurred, and such fractures were dominating for specimens roughened with a diamond disc prior to application (Figure 2). An example of cohesive fracture in porcelain is shown in Figure 3.

## DISCUSSION

To ensure the best prognosis for composite repair of feldspar ceramic chipping, it is crucial to determine which combination of surface treatment and bonding method provides the highest bond strength between the two materials and which of the steps is most important. In the present study, two surface treatments and two bonding methods were examined to assess their impact on bond strength. Although numerous studies have been conducted in this area, no consensus has been reached on whether surface treatment or bonding method holds greater significance [11]. The findings presented here suggest that the bonding method used is more critical for bond strength than the type of surface treatment. This is evident as specimens treated with the same adhesive system, but different surface manipulations, achieved similar results in shear bond strength testing, with only minor variations. Therefore, the results indicate that application of HF in patients' mouths is unnecessary and that roughening of the surface may be sufficient to achieve good bond strength. In restorative dentistry, a bond strength of 15–20 MPa is considered satisfactory [24]. In the present study, group R‐SA and group E‐SA achieved a mean bond strength of over 30 MPa. Thus, both types of surface treatment result in satisfactory and clinically significant bonding. This conclusion is supported by a systematic review by Nogueira et al. [11], who examined surface treatment methods of feldspathic porcelain in relation to bond strength. They concluded that bur roughening is a viable alternative to HF etching. Utilizing burs over HF is advantageous in practice regarding time management and patient safety, with the latter being particularly important because of the known toxicity risks associated with the use of HF [9].

Previous studies have shown varying results for bond strength with different surface treatments [6, 10, 11]; however, few distinctly separate the significance of surface treatment from that of bonding method, often reporting combined results instead, which complicates direct comparisons.

In the present study, the fracture mode results aligned with the shear bond strength results, indicating that surface treatment is less critical than the adhesive for achieving a strong bond between feldspathic porcelain and composite resin. This is reflected by the finding of significant differences in fracture mode between groups with the same surface treatment but different bonding methods. Conversely, similar distributions of adhesive and cohesive fractures, with only minor deviations, were found in groups with the same bonding method but different surface treatments. Group R‐SA displayed a lower percentage of adhesive fractures (30%) than group E‐SA (50%). These discrepancies suggest that the type of surface treatment might influence the bond achieved between feldspathic porcelain and composite resin, favouring surface roughening. This contrasts with the results of a study published by Özcan et al. [25], who reported a high frequency (70%) of adhesive fractures when the ceramic surface was roughened with a diamond bur prior to repair with composite resin. These differing results might be attributed to the grit of the diamond bur used by Özcan et al., which was referred to as fine, and the grit of the P220 rotating diamond disc used in our study (chosen to correspond with a medium‐grit dental bur). Also in contrast to our results, in a study by Valian et al. [26] no adhesive fractures were observed when the repair surface was etched with HF and different universal adhesives were applied, with or without a separate silane primer. These authors observed a high frequency of mixed fractures, but did not further specify the definition of this fracture type and the adhesive part of it.

The potential reason for the lesser importance of surface treatment compared with bonding method, observed in our study, may be that both HF and roughening with a P220 diamond disc created sufficient surface roughness for adequate micromechanical retention. Etching with HF increases surface roughness by dissolving the amorphous phase of feldspathic porcelain, providing a larger surface area for bonding. Numerous studies describe how etching with HF is crucial for achieving good bond strength [11, 27]. In contrast, few studies explain the effect of roughening and grit on surface roughness, making it challenging to compare the impact of the two surface treatments on roughness. Another explanation could be that micromechanical retention is less significant than chemical bonding, as indicated by our results.

The application of silane is a crucial step in optimizing bond strength between feldspathic porcelain and composite resin [11]. Nonetheless, our study showed significant differences in bond strength between the use of a universal adhesive and an adhesion protocol involving separate steps with a silane primer and an adhesive, favouring the latter, despite both containing silane. Therefore, we recommend the use of separate silane primer and adhesive for composite resin repairs of feldspar porcelain rather than the use of universal adhesives (typically used for composite resin fillings). This claim is supported in a study by Noda et al. [28], in which it was concluded that the universal adhesive had significantly lower bond strength to ceramic materials, including feldspathic porcelain, compared with other more ceramic‐specific two‐step adhesives. Similarly, in a study by Almaskin et al. [23], shear bond strength was compared across groups treated with a silane‐containing universal adhesive or with a separate silane primer and adhesive, and the findings favoured the use of separate primer and adhesive, regardless of application time. The suggested reason for this variance is the different silane monomers in these systems: universal adhesives contain a less effective silane monomer with a shorter duration of stability compared with those in separate silane primers. The difference in effectiveness of silane monomers in a universal adhesive and separate silane primer can be explained by the varying pH levels of the products. Silane primers used in dentistry typically have a pH between 4 and 5, where they are most stable. Conversely, when silane monomers are added to an adhesive with pH levels as low as 2, their effectiveness and stability are compromised as the monomers hydrolyse in such acidic solutions, altering their form and properties [15]. The universal adhesive used in the present study has a pH of 2.7 [29], and this possibly explains the weaker bond strength of this adhesive compared with the bond strength generated following use of a separate silane primer and adhesive. Finally, it should also be considered that the concentration of silane is lower in universal adhesives than in separate silane primers, and further, that an interaction between silane and other monomers in the universal adhesive might affect the bond strength [26].

The fracture mode distribution further supports this, as 70% and 50% cohesive fractures in the feldspathic porcelain were observed in the two groups in which separate primer and adhesive were used. In the groups in which universal adhesive was used, all specimens tested showed adhesive fractures between feldspathic porcelain and composite resin. Cohesive fractures in the feldspathic porcelain indicate that the bond to composite resin remained intact, suggesting that bond strength exceeded the fracture strength of the ceramic. However, during roughening of the surface with a P220 diamond disc, the ceramic may have been weakened, resulting in lower cohesive strength affecting the fracture mode. Regardless, separate silane primer and adhesive demonstrated significantly better bond strength than the universal adhesive.

Our study is limited to composite resin repairs where the entire fracture surface is feldspathic porcelain. This technique is unlikely to yield satisfactory outcomes for adhesive fractures between core material and feldspathic porcelain, as silane does not bond chemically to metal or zirconia. For scenarios in which a smaller area of feldspathic porcelain is exposed in adhesive fractures, specific primers for the core material should be utilized [30, 31].

Although our study presents a clear result in favour of the use of separate silane primer and adhesive, the method requires consideration. Thermocycling was used to simulate aging resulting from the consumption of hot and cold food and drinks; however, the translatability of thermocycling to clinical practice remains a matter of debate [32, 33]. Another crucial factor is that the magnitude and direction of forces applied during shear bond strength testing may not directly correlate to the forces experienced by a restoration in the oral cavity. Therefore, the applicability of the results to clinical practice warrants discussion. The study was conducted under controlled conditions, whereas clinical practice involves variables such as saliva, which may contaminate the fracture surface, impacting the bond between feldspathic porcelain, adhesive, and composite resin. Nonetheless, using a rubber dam can greatly mitigate this, ensuring effective moisture control [34]. Additionally, the number of procedural steps may affect outcomes; more steps might increase contamination risk of the fracture surface, thus rendering the procedure more technique sensitive. A systematic review and meta‐analysis of randomized controlled clinical trials [35] evaluated if reduced bonding procedure time, through fewer steps, held clinical significance for retention. The review concluded insignificance between one‐step and two‐step procedures regarding retention, suggesting procedural equivalence. Nonetheless, the number of steps might still impact the results given the enhanced effectiveness of silane monomers when separated from adhesive [15, 36]. Such dynamics may change with technological advancements in dental materials. Investigating the effectiveness of new materials would be beneficial for future research.

## AUTHOR CONTRIBUTIONS

**Conceptualization**: Mina Aker Sagen, Bjørn Einar Dahl; **Formal analysis**: Mina Aker Sagen; **Investigation**: Bjørk Eriksen, Kaja Moland; **Methodology**: Mina Aker Sagen, Bjørn Einar Dahl; **Writing—original draft**: Mina Aker Sagen, Bjørk Eriksen, Kaja Moland; **Writing—reviewing and editing**: Mina Aker Sagen, Bjørn Einar Dahl; **Project administration**: Mina Aker Sagen, **Supervision**: Bjørn Einar Dahl.

## CONFLICT OF INTEREST STATEMENT

The authors declare no conflict of interest.
